# Impact of physiological ability and surgical stress score on postoperative complications in patients undergoing surgery for non-muscle-invasive bladder cancer

**DOI:** 10.1097/MD.0000000000047699

**Published:** 2026-03-13

**Authors:** Junchao Bai

**Affiliations:** a Urology Department, The Fourth Affiliated Hospital of Zhejiang University School of Medicine, Jinhua, Zhejiang, China.

**Keywords:** complication, non-muscle-invasive bladder cancer, physiological ability, predictor, surgical stress score

## Abstract

This study aims to evaluate the predictive value of the physiological ability and surgical stress (E-PASS) scoring system for postoperative complications in patients undergoing surgery for non-muscle-invasive bladder cancer (NMIBC). This retrospective study included patients with NMIBC who underwent transurethral resection of bladder tumor. The E-PASS score was calculated for each patient. Postoperative in-hospital complications were recorded and graded according to the Clavien–Dindo classification. Logistic regression analysis was used to identify independent risk factors, and receiver operating characteristic curves were constructed to assess predictive performance. Significant differences were observed between the two groups in intraoperative blood loss, tumor differentiation grade, and the E-PASS scores (all *P* < .05). Multivariate analysis identified poor differentiation grade (odds ratio [OR] = 1.625), greater intraoperative blood loss (OR = 1.239), preoperative risk score (OR = 1.442), surgical stress score (OR = 1.376), and comprehensive risk score (CRS; OR = 1.272) as independent risk factors for in-hospital complications (all *P* < .05). Receiver operating characteristic analysis demonstrated high predictive accuracy for the E-PASS components: the area under the curve was 0.926 for preoperative risk score (sensitivity 85.71%, specificity 85.77%), 0.915 for surgical stress score (sensitivity 71.43%, specificity 89.29%), and 0.942 for CRS (sensitivity 78.57%, specificity 89.29%). Furthermore, at 24 and 36 months post-surgery, the recurrence rate was significantly lower in the non-complication group compared to the complication group (*P* < .05). The E-PASS scoring system, particularly the CRS, is a useful tool for predicting postoperative complications in patients undergoing surgery for NMIBC.

## 1. Introduction

Non-muscle-invasive bladder cancer (NMIBC) is also known as superficial bladder cancer. The neoplasm of this cancer is confined to subepithelial connective tissue and does not invade the muscular layer.^[1]^ NMIBC accounts for 70% of primary bladder tumors, including 70% in TA, 20% in T1 and 10% in TIS.^[2]^ At present, the treatment of NMIBC is mainly based on operation. In recent years, some new concepts have been put forward and there are also certain controversies.^[3]^ Transurethral resection of bladder tumor is a commonly used surgical method, which has the advantages of less trauma, quick recovery, simple operation, less bleeding, and preservation of the bladder. However, there are residual cancer and pathological staging deviations after operation, with high recurrence rate and risk of progression to muscular infiltration.^[4,5]^ Therefore, it is necessary to study tools for predicting the occurrence of postoperative in-hospital complications in NMIBC patients.

The estimation of physiological ability and surgical stress (E-PASS) scoring system is mainly used to assess the patient’s physiological status and the stress response to operation on the body. Through the physiological ability and surgical stress score (SSS), doctors can obtain a comprehensive understanding of patients’ physical conditions and predict the risks and prognosis of operation.^[6]^ At the same time, these scores can also help physicians to formulate better treatment plans and ensure that patients receive optimal care and treatment before, during and after operation.^[7]^ Current studies have shown that E-PASS scores show a great effect in assessing the postoperative mortality of patients with colorectal cancer and biliary calculus.^[8]^

However, the prognostic value of E-PASS for postoperative nosocomial complications in NMIBC patients remains unclear. To accurately describe the physical condition of these patients, a practical scoring system that includes assessment of their preoperative condition and their response to invasive surgical procedures is essential. Therefore, we retrospectively evaluate the predict the E-PASS score system for predicting the risk of postoperative nosocomial complications in NMIBC patients.

## 2. Materials and methods

### 2.1. Study design and participants

This study was approved by the Ethics Committee of The Fourth Affiliated Hospital of Zhejiang University School of Medicine.120 NMIBC patients admitted to our hospital from Jan 2020 to Aug 2023 were retrospectively selected. Inclusion criteria: all patients met the clinical diagnosis of NMIBC; patients with primary bladder cancer; patients who meet the surgical indications, and with complete clinical data. Exclusion criteria: with other malignant tumors; with mental disorders; with distant metastasis; and with other surgical contraindications.

This study was approved by the Ethics Committee of our hospital in accordance with regulatory and ethical guidelines pertaining to retrospective research studies. Informed consent was waived for this retrospective study due to the exclusive use of de-identified patient data, which posed no potential harm or impact on patient care.

### 2.2. Setting

Patients were divided into complication group (n = 40) and non-complication group (n = 80) according to whether they had postoperative in-hospital complications. Clavien–Dindo classification criteria were used to evaluate the incidence of postoperative complications in the 2 groups. According to this criterion, levels 2 to 4 of adverse events are defined as postoperative complications, as these levels of events require additional medical intervention, including, but not limited to, medication. Grade 1 and below adverse events were excluded from this analysis because they usually did not require additional drug treatment.^[9]^ Preoperative risk score (PRS), SSS and comprehensive risk score (CRS) were calculated by E-PASS system. The E-PASS scoring system equation is:


$$\begin{array}{l} \begin{array}{lll} & PRS=-0.0686+0.00345*X1+0.323*X2+0.205*X3\; & +0.153*X4+0.148*X5+0.0666*X6\; \end{array} \end{array}$$

X1 refers to the age of the patient, X2 refers to the presence (1) or absence of (0) severe heart disease, X3 refers to the presence (1) or absence (0) of severe lung disease, X4 refers to the presence (1) or deletion (0) diabetes, X5 is the performance status index (0–4), and X6 is the physiological status classification of the American Medical Association (1–5).


$$\begin{array}{l} \begin{array}{lll} & SSS=-0.342+0.0139*X1+0.0392*X2/60\; & +0.352*X3\; \end{array} \end{array}$$

X1 is the blood loss/ body weight (g/ kg), X2 is the operation time (h), X3 is the range of skin incision, (laparoscopic or video-assisted thoracoscopic surgery 1, laparotomy or thoracotomy 2, laparotomy and thoracotomy 3)


$$\begin{array}{l} CRS=-0.328+0.936*PRS+0.976*SSS \end{array}$$

Surgical procedures: Minimally invasive surgery: transurethral resection of bladder tumor, which uses plasma, electroresection or laser to complete the operation in the cavity without surgery.

During the operation, the electrosurgical endoscope enters the bladder through the urethra, finds the focus of the bladder, and uses an energy platform such as laser or plasma to remove the tumor inside the bladder. After resection, some chemotherapeutic drugs were sprayed locally to prevent tumor recurrence. This operation is suitable for non-myometrial invasive bladder cancer, that is, the root of bladder cancer only invades the mucous membrane and submucosa, but not the bladder muscle.

### 2.3. Observation indicators

Univariate analysis of influencing factors of postoperative hospital complications in patients with NMIBC. Multivariate LOGISTICS regression analysis of influencing factors of postoperative hospital complications in patients with NMIBC. Receiver operating characteristic (ROC) analysis was used to analyze the value of physiological ability and SSS in predicting postoperative hospital complications in NMIBC patients. The recurrence rates of the 2 groups were compared.

### 2.4. Statistical analysis

The collected experimental data were analyzed using SPSS 27.0 (IBM Corp., Armonk). Measurement data conforming to normal distribution in the experimental data were expressed as *X̅* ± *S*, and independent-samples *t* test was used for comparison. Enumeration data were expressed as number of cases or rate. Univariate and binary logistics regression analyses were performed using χ^2^ test or Fisher’s exact method to analyze the influencing factors of postoperative in-hospital complications in patients with NMIBC. The ROC curve was used to evaluate the predictive value of physiological ability and SSS on postoperative in-hospital complications in patients with NMIBC, and *P* < .05 was considered statistically significant.

## 3. Results

### 3.1. Results of single factor analysis

Clinicopathological characteristics of patients are summarized in Table 1. Age, sex, body mass index, tumor diameter, number of Tumors, diabetes and hypertension in the 2 groups were not significantly different, but the intraoperative blood loss, degree of differentiation and E-PASS were significantly worse in complication group (*P* < .05).

**Table 1:** Results of single factor analysis.

Factors		Complication group (n = 40)	Non-complication group (n = 80)	*t/χ* ^2^	*P* value
Age (yr)		53.56 ± 5.23	53.77 ± 5.27	0.206	.837
Sex	Male	21	45	0.152	.927
	Female	19	35		
BMI (kg/m^2^)		21.87 ± 2.12	21.97 ± 1.97	0.256	.799
Tumor diameter (cm)		3.71 ± 1.29	3.77 ± 1.22	0.249	.804
Number of Tumors (No.)		1.41 ± 0.77	1.43 ± 0.81	0.130	.897
Diabetes	Yes	12	20	3.225	.199
	N/A	28	60		
Hypertension	Yes	10	22	0.085	.958
	N/A	30	58		
Intraoperative blood loss (mL)		142.81 ± 20.87	133.25 ± 20.22	2.416	.017
Degree of differentiation	Well-differentiated	17	17	8.583	.035
	Moderately differentiated	12	20		
	Poorly differentiated	11	43		
E-PASS	PRS	0.33 ± 0.12	0.30 ± 0.02	2.185	.031
	SSS	0.49 ± 0.11	0.32 ± 0.10	8.489	<.0001
	CRS	0.47 ± 0.14	0.29 ± 0.07	9.409	<.0001

BMI = body mass index, CRS= comprehensive risk score, E-PASS = estimation of physiological ability and surgical stress, PRS = preoperative risk score, SSS = surgical stress score.

### 3.2. Results of multivariate logistics regression analysis

The degree of differentiation, intraoperative bleeding volume, PRS, SSS, and CRS were assigned as independent variables (actual values substituted), and the outcome of trial delivery was used as dependent variable for analysis (occurred complications = 1, did not occur = 0). The results of multivariate logistics regression analysis showed that the degree of differentiation, intraoperative bleeding volume, PRS, SSS, and CRS were independent influencing factors for postoperative in-hospital complications in patients with NMIBC (*P* < .05), as shown in Table 2.

**Table 2:** Multivariate logistics regression analysis of influencing factors of postoperative hospital complications in patients with nonmuscular invasive bladder cancer.

Factors	*Β*	SE	Ward	OR	95% CI	*P* value
Degree of differentiation	0.486	0.195	6.199	1.625	1.109–2.381	<.001
Intraoperative bleeding volume	0.214	0.103	4.329	1.239	1.013–1.516	<.001
PRS	0.366	0.168	4.747	1.442	1.037–2.004	<.001
SSS	0.319	0.135	5.590	1.376	1.056–1.793	<.001
CRS	0.241	0.107	5.056	1.272	1.031–1.569	<.001

CI = confidence interval, CRS= comprehensive risk score, OR = odds ratio, PRS = preoperative risk score, SE = standard error, SSS = surgical stress score.

### 3.3. Results of ROC analysis

**Figure 1.**
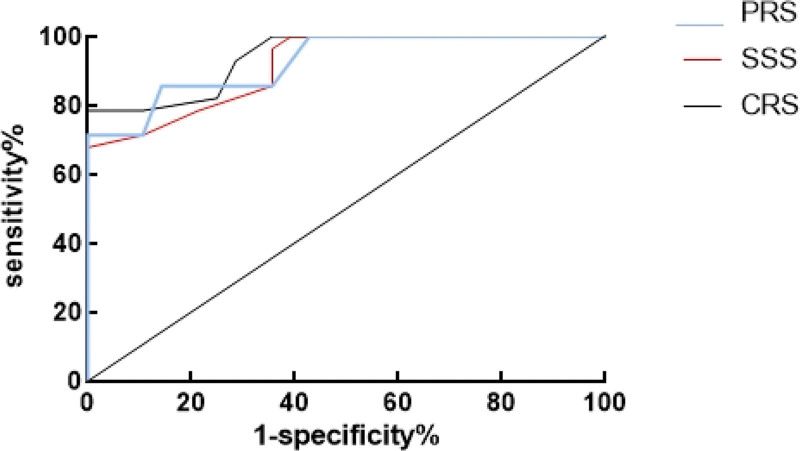
Results of ROC analysis. CRS= comprehensive risk score, PRS = preoperative risk score, ROC = receiver operating characteristic, SSS = surgical stress score.

An analysis utilizing ROC curves revealed that the area under the curve (AUC) for the PRS prediction model was 0.926, accompanied by a standard error of 0.033 and a 95% confidence interval (CI) ranging from 0.8617 to 0.9903. The optimal cutoff value was determined to be 0.71, yielding a sensitivity of 85.71% and a specificity of 85.77%. Similarly, for the SSS prediction model, the AUC was calculated as 0.915, with a standard error of 0.035 and a 95% CI spanning from 0.8453 to 0.9838. The optimal threshold was identified as 0.61, corresponding to a sensitivity of 71.43% and a specificity of 89.29%. Finally, the CRS prediction model exhibited an AUC of 0.942, accompanied by a standard error of 0.028 and a 95% CI ranging from 0.8871 to 0.9968. The optimal threshold for this model was 0.68, resulting in a sensitivity of 78.57% and a specificity of 89.29%, as shown in Figure 1.

### 3.4. Complication result

At 24 and 36 months after operation, the recurrence rate in the non-complication group was significantly lower than that in the complication group (*P* < .05), see Table 3.

**Table 3:** Complication result.

	Complication group	Non-complication group	*χ* ^2^	*P* value
Number of cases	40	80		
3 m after operation	1 (2.50%)	0 (0.00%)	2.017	.365
6 m after operation	2 (5.00%)	0 (0.00%)	4.068	.131
12 m after operation	4 (10.00%)	1 (1.25%)	1.694	.429
24 m after operation	7 (17.50%)	2 (2.50%)	8.649	.013
36 m after operation	9 (22.50%)	4 (5.00%)	8.454	.015

## 4. Discussion

In the surgical treatment of NMIBC patients postoperative complications are a problem that cannot be ignored. To better understand the influencing factors of postoperative complications and effectively prevent and treat patients, this study conducted an in-depth study on the E-PASS score on postoperative in-hospital complications in NMIBC patients. It is important to find that physiological ability and SSS significantly affect the incidence of postoperative hospital complications in patients with NMIBC. The innovation is that it combines the evaluation of physiological ability and the quantitative analysis of surgical stress, which provides a new perspective and method for predicting and reducing postoperative complications in such patients. This finding not only helps doctors assess the risk of patients more accurately before operation, but also provides a reference for the development of personalized treatment plans.

There were significant differences in intraoperative blood loss, degree of differentiation and E-PASS between the 2 groups (*P* < .05). After analyzing the reasons, the intraoperative bleeding amount may be related to the surgical operation and tumor invasion degree. If there is more bleeding during the operation, it may mean that the operation is difficult or the tumor invades extensively, which will increase the risk of postoperative complications.^[10]^ Therefore, for patients with a large amount of intraoperative bleeding, doctors should pay more attention to the postoperative recovery and take corresponding measures to prevent complications.^[11,12]^ The degree of differentiation is also one of the important factors affecting postoperative in-hospital complications. In general, the less differentiated and more malignant tumors are associated with a correspondingly increased risk of postoperative complications for patients.^[13]^ Therefore, for patients with low differentiation, physicians should strengthen postoperative monitoring and care and develop personalized treatment regimens to reduce the risk of complications.^[14]^ The degree of differentiation, intraoperative bleeding volume, PRS, SSS, and CRS were further assigned as independent variables (actual values substituted), and the outcome of trial delivery was used as dependent variable for analysis (complications = 1, no complications = 0). Multi-factor result showed that the degree of differentiation, intraoperative bleeding volume, PRS, SSS, and CRS were independent influencing factors for postoperative in-hospital complications in NMIBC patients (*P* < .05), further demonstrating the influence of intraoperative bleeding volume, degree of differentiation and E-PASS on postoperative complications in NMIBC patients.^[15]^

Previous studies by Kasap,^[16]^ et al showed that the critical value of E-PASS CRS for predicting postoperative complications was −0.2996 (AUC = 0.706%, CI: 0.629–0.783%, *P *< .001), indicating that E-PASS score can predict postoperative complications after laparoscopic nephrectomy. In the past, there were few studies on the prediction of complications of NMIBC, but E-PASS was applied to it, and the results showed that the application value of E-PASS in predicting postoperative in-hospital complications in NMIBC patients was further analyzed. ROC results showed that the AUC of prediction of postoperative hospital complications in NMIBC patients was lower area: 0.926, standard error: 0.033, (95% CI: 0.8617–0.9903), best truncation value: 0.71, sensitivity: 85.71, 85.77 area under CRS: 0.915, standard error: 0.035, (95% CI: 0.8453–0.9838), optimum truncation value: 0.61, sensitivity: 71.43, 89.29, area under CRS: 0.942. Standard error: 0.028, (95% CI: 0.8871–0.9968), optimal truncation value: 0.68, sensitivity: 78.57, specificity: 89.29. The optimal cutoff value was 0.68, and the sensitivity and specificity were 78.57 and 89.29 respectively. The results showed that PRS, SSS, and CRS had high accuracy in predicting postoperative in-hospital complications. Specifically, CRS was the best predictor with an AUC of 0.942. The predictive value of the E-PASS was demonstrated.

In addition, the comparison of postoperative recurrence rates showed that the recurrence rate in the uncomplicated group at 24 and 36 months after operation was significantly lower than that in the complications group. The reason may be that the occurrence of complications affected the recovery and treatment effect of patients. Postoperative complications may lead to weakness, decreased immunity and susceptibility to infection and recurrence in patients.^[17,18]^ In addition, the occurrence of complications may also affect the psychological state and quality of life of patients, resulting in depressed mood, anxiety, depression, etc. These adverse emotions will also affect the rehabilitation and treatment efficacy of patients.^[19]^ Physicians should pay attention to postoperative care and rehabilitation of patients, formulate personalized treatment plans and nursing plans, strengthen psychological support and health education for patients, and improve self-management and health awareness of patients, to reduce the incidence and recurrence rate of postoperative complications.

Although this study reveals the value of physiological ability and SSS in predicting postoperative hospital complications in patients with NMIBC, there are still some shortcomings. First of all, the sample size may be relatively small, which may limit the wide applicability and statistical effectiveness of the research results. In addition, this study mainly focuses on in-hospital complications, and the assessment of long-term complications may not be comprehensive. In future research, we should further expand the sample size, consider the potential factors more comprehensively, and evaluate the long-term complications in order to provide more accurate and comprehensive conclusions.

## 5. Conclusion

The E-PASS scoring system, especially the CRS, can serve as a practical and effective tool for identifying NMIBC patients at increased risk of postoperative complications, thereby supporting perioperative risk stratification and clinical decision-making.

## Author contributions

**Conceptualization:** Junchao Bai.

**Data curation:** Junchao Bai.

**Formal analysis:** Junchao Bai.

**Funding acquisition:** Junchao Bai.

**Investigation:** Junchao Bai.

**Writing – original draft:** Junchao Bai.

**Writing – review & editing:** Junchao Bai.
